# Deep Multi-Feature Transfer Network for Fourier Ptychographic Microscopy Imaging Reconstruction †

**DOI:** 10.3390/s22031237

**Published:** 2022-02-06

**Authors:** Xiaoli Wang, Yan Piao, Jinyang Yu, Jie Li, Haixin Sun, Yuanshang Jin, Limin Liu, Tingfa Xu

**Affiliations:** 1 Information and Communication Engineering, Electronics Information Engineering College, Changchun University of Science and Technology, Changchun 130022, China; wangxl@ccu.edu.cn; 2 Electrical and Electronic Teaching Center, Electronics Information Engineering College, Changchun University, Changchun 130022, China; 190401067@mails.ccu.edu.cn (J.Y.); haixin_s@hotmail.com (H.S.); 200401080@mails.ccu.edu.cn (Y.J.); 200401097@mails.ccu.edu.cn (L.L.); 3 School of Optics and Photonics, Beijing Institute of Technology, Beijing 100081, China; ciom_xtf1@bit.edu.cn

**Keywords:** Fourier ptychographic microscopy, transfer learning, neural network, deep multi-feature

## Abstract

Fourier ptychographic microscopy (FPM) is a potential imaging technique, which is used to achieve wide field-of-view (FOV), high-resolution and quantitative phase information. The LED array is used to irradiate the samples from different angles to obtain the corresponding low-resolution intensity images. However, the performance of reconstruction still suffers from noise and image data redundancy, which needs to be considered. In this paper, we present a novel Fourier ptychographic microscopy imaging reconstruction method based on a deep multi-feature transfer network, which can achieve good anti-noise performance and realize high-resolution reconstruction with reduced image data. First, in this paper, the image features are deeply extracted through transfer learning ResNet50, Xception and DenseNet121 networks, and utilize the complementarity of deep multiple features and adopt cascaded feature fusion strategy for channel merging to improve the quality of image reconstruction; then the pre-upsampling is used to reconstruct the network to improve the texture details of the high-resolution reconstructed image. We validate the performance of the reported method via both simulation and experiment. The model has good robustness to noise and blurred images. Better reconstruction results are obtained under the conditions of short time and low resolution. We hope that the end-to-end mapping method of neural network can provide a neural-network perspective to solve the FPM reconstruction.

## 1. Introduction

The development of microscopic imaging technology has opened the door for human beings to explore the microscopic world, microscope has become an indispensable tool in the field of life sciences, and every progress in microscopic imaging technology has promoted the development of various fields. Although the microscopic imaging technology has made great progress in recent years, the imaging mechanism of its technology has not fundamentally changed. It is still based on the imaging principle of traditional lens, that is, the imaging mode of what is seen and what is obtained. Although the traditional imaging mechanism is simple and easy to implement, it still faces many bottleneck problems. It is difficult to solve the challenges brought by the new application requirements [1,2]. Fourier ptychographic microscopy is an imaging technology with large field of view, high resolution and quantitative phase calculation developed in recent years [3,4,5]. This technology uses an optical microscope and LED lighting array [6], which combines phase retrieval algorithm to recover the high-resolution intensity and phase of the sample [7,8]. Fourier ptychographic microscopy is based on the intensity information acquired in the spatial domain and a fixed mapping relationship in the frequency domain to iterate alternately. In the spatial domain, the amplitude is constrained by the low-resolution image, and in the frequency domain, the spectrum support domain is constrained by the CTF (Coherent Transfer Function). It overcomes the problems brought by the traditional lens imaging mechanism and solves the contradiction between resolution and large field of view from the perspective of calculation. Moreover, the phase retrieval mechanism can not only reconstruct the intensity image, but also generate the quantitative phase image of the sample at the same time.

Since the FPM was proposed, various methods have been proposed to improve the reconstruction performance and reduce the computational complexity of FPM. In terms of system setting, pupil aberration correction [9,10], LED misalignment correction [11,12] and more efficient LED module design [13,14] are proposed to improve reconstruction quality. In terms of reconstruction methods, multiplexing strategy is introduced [15,16] to accelerate the image acquisition process, and optimization theories such as Wirtinger-flow, adaptive step-size strategy, and convex relaxation [17] are proposed to improve reconstruction speed and robustness. Recently, with the rapid development of convolutional neural networks, people have introduced a convolutional neural network (CNN) into the FPM reconstruction process and can learn the mapping from low resolution intensity input to high resolution complex output to solve the problem of FPM reconstruction. Jiang et al. [18] modeled FPM through convolutional neural network and reconstructed FPM through back propagation. Zhang et al. [19] introduced Zernike aberration recovery and total variation constraints based on neural network to ensure the quality of FPM reconstruction and at the same time to correct aberration. The above work is still based on iterative algorithms and does not give full play to the advantages of deep learning. Deep learning finds the linear mapping between input and output by building Deep Neural Networks (DNN) [20,21], and obtains the weight information of Deep Neural Networks in the case of mass data training to solve the reconstruction quality problem. In recent years, DNN-based methods have achieved good results in image reconstruction [22,23,24,25] and phase recovery [26,27], and some researchers have proposed the FPM reconstruction method using DNN [28,29,30], which performs well in high-resolution image reconstruction. One of the authors of this paper proposed a fmcnn algorithm at the 2021 International Conference on electronic information engineering and computer science. This algorithm is a Fourier superposition micro reconstruction method based on multi convolution network feature fusion, which realizes image reconstruction from low resolution to high resolution. Additionally, it constantly adjusts the super parameters of the network model to make it have better reconstruction effect and robustness to noisy images [31].

In this paper, we focus on solving the FPM reconstruction quality problem by deep learning. Using ResNet50 [32], Xception [33] and Densenet121 [34] transfer learning networks to extract image features. Cascaded feature fusion strategy and pre-upsampling reconstruction network are used to build deep multi-feature transfer network (DMFTN) for Fourier ptychographic microscopy imaging model to solve the problem of FPM reconstruction. DMFTN fully extracts the features at each level in the input original low-resolution image, preserves and amplifies the information features, which makes the information transmission more complete and efficient. DMFTN uses the complementarity of the extracted features of different migratory learning networks to increase the redundancy of the extracted feature information and better reconstructs the high-resolution image, and adopts the pre-upsampling reconstruction. DMFTN is capable of reconstructing high-resolution complex amplitudes of many types of samples. In order to train the reconstruction network, the acquired FPM intensity images are synthesized into two-channel complex amplitudes such as network model inputs, and the reconstructed outputs of network model outputs are also two-channel complex amplitudes. The 400 animal tissue images were acquired and used to build a simulation dataset through the FPM imaging model. The simulation data set is divided into training data set and test data set to train and test the proposed network model.

## 2. Related Works

The FPM consists of two main components, the imaging model and the reconstruction model. The imaging model is an objective description of the imaging acquisition process, representing the process of plane waves passing through the microscope and imaged on the sensor. The reconstruction model is the reconstruction of the high-resolution complex amplitude process using the low-resolution image acquired by the sensor combined with the phase recovery algorithm.

### 2.1. Imaging Model of Fourier Ptychographic Microscopic

**Figure 1:**
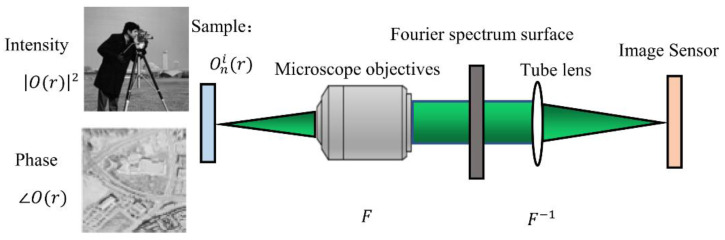
Schematic diagram of FPM imaging model.

FPM is a computational method that combines the original low-resolution data into high-resolution and wide-field images. The imaging process is shown in Figure 1.

In the process of imaging, the objective lens and cylinder lens in the system carry out two Fourier transforms of the light waves of the object in turn. First of all, the sample function is expressed by the light emitted by LED. When the light emitted by the sample is illuminated on the sample, the light wave is equivalent to a monochromatic plane wave that is oblique incidence. The sample function interacts with the tilted plane waves $e^{{\mathrm{ik}}_{\mathrm{xi}}x},{\;e}^{{\mathrm{ik}}_{\mathrm{yi}}y}$ to produce the radiation field $e\left(r\right)=o\left(x,y\right)e^{{\mathrm{ik}}_{\mathrm{xi}}x}e^{{\mathrm{ik}}_{\mathrm{yi}}y}$, where $k_{\mathrm{xi}}$ and $k_{\mathrm{yi}}$ are the wavenumber in the x and y directions, respectively. For the case where the nth LED is lit, the wave vector of the incident light can be expressed as:

(1) $$k_{n}=\left(\frac{{\sin\alpha }_{\mathrm{xn}}}{\lambda },\frac{{\sin\alpha }_{\mathrm{yn}}}{\lambda }\right)\;\left(n=1,2,\ldots ,N_{\mathrm{LED}}\right)$$

where $\left(\alpha_{\mathrm{xn}},\alpha_{\mathrm{yn}}\right)$ represents the incident angle of the illuminating light wave, λ represents the wavelength of the light, and n represents the number of LED. When n=1, it indicates positive incidence. Assuming that the intensity of the incident light is 1 and the initial phase is 0, then the incident light can be expressed as $\exp\left({\mathrm{jk}}_{n}r\right)$ when the incident wave vector is $k_{n}$. After passing through the sample, the plane wave emits light $e\left(r\right)=o\left(x,y\right)e^{{\mathrm{ik}}_{\mathrm{xi}}x}e^{{\mathrm{ik}}_{\mathrm{yi}}y}$. The objective lens transforms the field by Fourier transform, so it is expressed as:

(2) $$F\left\{e\left(r\right)\right\}=F\left\{o\left(x,y\right)e^{{\mathrm{ik}}_{\mathrm{xi}}x}e^{{\mathrm{ik}}_{\mathrm{yi}}y}\right\}=O\left(k-k_{n}\right)$$

This is equivalent to moving the original spectral center of the sample to the $k_{n}$ position.

The spectrum is filtered by the coherent transfer function H(k) of the objective lens, and the components of the spectrum that can be accepted by the optical system after filtering are:

(3) $$G_{n}\left(k\right)=O\left(k-k_{n}\right)H\left(k\right)$$

The obtained spectrum is filtered by pupil aperture low-pass filter, and the cutoff frequency is NA 2π/λ, where NA is the numerical aperture and λ is the optical wavelength. Through coordinate transformation, Equation (3) can be written as follows:

(4) $$G_{n}\left(k+k_{n}\right)=O\left(k\right)H\left(k+k_{n}\right)$$

Finally, the tube lens carries out the second Fourier transform of the filtered spectrum O(x,y), and the sensor captures the final intensity image I(x,y).

The outgoing light wave is imaged on the image plane and is received by the image sensor and converted into a digital signal.

(5) $$I_{\mathrm{nc}}\left(x,y\right)={\left|g_{n}\left(r\right)\right|}^{2}={\left|F^{-1}\left\{G_{n}\left(k+k_{n}\right)\right\}\right|}^{2}={\left|F^{-1}\left\{O\left(k\right)H\left(k+k_{n}\right)\right\}\right|}^{2}$$

where $g_{n}\left(r\right)$ is the complex amplitude of reaching the image plane. As the image sensor only responds to the intensity of light, the final image is $I_{\mathrm{nc}}\left(r\right)$, and the subscript c represents the actual value collected.

### 2.2. Reconstruction Model of Fourier Ptychographic Microscopy

The reconstruction process of Fourier ptychographic microscopy combines the concepts of phase recovery and synthetic aperture. Phase recovery is first used to solve the problem of phase loss in electronic diffraction. In 1971, Gerchberg and Saxton first proposed their phase iterative recovery algorithm, so their phase iterative recovery algorithm is also known as the GS algorithm. It uses the Fourier transform relationship between the spatial domain and the frequency domain, and adds constraints in the spatial domain and the frequency domain to iterative alternately, so as to continuously approximate and converge to the real complex amplitude of the object. FPM is committed to iteratively reduce the difference between the low-resolution amplitude corresponding to the guessed object function and the actual low-resolution amplitude, which can be seen as a nonconvex optimization problem.

The concrete steps of the reconstruction process are:

1. Initial guess

At the beginning of the algorithm, it is necessary to initially guess the high-resolution object function and the coherent transfer function. In the case of sufficient data redundancy, FPM can converge no matter what kind of guess is used, such as complete guess, random guess, and the initial guess has no effect on the final result. In order to improve the convergence speed and reduce the number of iterations, the low-resolution image under normal incidence is often used as the initial strength guess of the object, and the initial phase guess of the object is generally initialized to zero. CTF carries on the initial guess according to the ideal CTF without aberration, as shown;

(6) $$O_{0}^{0}\left(k\right)=F\left\{\sqrt{B\left(I_{1c}\left(r\right)\right)}\exp\left(\mathrm{jk}\cdot \varphi_{0}\right)\right\}H\left(k\right)$$

where${\;O}_{0}^{0}\left(k\right)$ denotes the initial value of the sample spectrum. B denotes the bilinear interpolation of the image for amplification. $\varphi_{0}$ denotes the initialized phase, which is generally taken as 0.

2. Low resolution light field in computational imaging face

Under the illumination of LED at the corresponding angle, the light wave field emitted by the object is Fourier transformed and then passes through the low pass of the optical system. The light wave field after the low pass reaches the imaging surface through an inverse Fourier transform. The corresponding low-resolution estimated light field is

(7) $$g_{n}^{i}\left(r\right)=F^{-1}\left\{G_{n}^{i}\left(k+k_{n}\right)\right\}=F^{-1}\left\{O_{n-1}^{i}\left(k\right)H\left(k+k_{n}\right)\right\}$$

where i represents the number of current iterations $\left(i=1,2,\ldots ,i_{\max}\right)$. n represents the different incidence angles $\left(n=1,2,\ldots ,N_{\mathrm{LED}}\right)$. $O_{n-1}^{i}\left(k\right)$ denotes the sample spectrum after the $i^{th}$ round update of ${\left(n-1\right)}^{\mathrm{th}}$ sub-spectrum, and $O_{0}^{i}\left(k\right)$ represents the initial value of the $i^{th}$ initial value of the spectrum updated in the first round, and${\;g}_{n}^{i}\left(r\right)$ denotes the complex amplitude estimate corresponding to the $n^{th}$ sub-spectrum in the $i^{th}$ update round.

3. Update the amplitude of the light field

Keeping the phase information of the low resolution estimated light field unchanged, the corresponding low resolution intensity image is used to update its amplitude information to obtain the updated low resolution estimated light field:

(8) $$\bar{g_{n}^{i}}=\sqrt{I_{\mathrm{nc}}\left(r\right)}\frac{g_{n}^{i}\left(r\right)}{\left|g_{n}^{i}\left(r\right)\right|}$$

where $\bar{g_{n}^{i}}$ denotes the updated sample complex amplitude for the $n^{\mathrm{th}}$ illumination, and $I_{\mathrm{nc}}\left(r\right)$ is the actual acquired low-resolution intensity image corresponding to the $n^{\mathrm{th}}$ illumination.

4. Spectrum of update function

The updated low-resolution estimated optical field is transformed into the frequency domain by Fourier transform $\bar{G_{n}^{i}}\left(k+k_{n}\right)=F\left\{\bar{g_{n}^{i}}\right\}$, and the object function spectrum in the subaperture is updated. Other regions remain unchanged:

(9) $$O_{n}^{i}\left(k\right)=O_{n-1}^{i}\left(k\right)\left[1-H\left(k+k_{n}\right)\right]+\bar{G_{n}^{i}}\left(k+k_{n}\right)H\left(k+k_{n}\right)$$

where $O_{n}^{i}\left(k\right)$ denotes the sample spectrum for the $n^{\mathrm{th}}$ sub-spectrum updated in the $i^{\mathrm{th}}$ round. The above formula indicates that only the part of the spectrum that is selected by $H\left(k+k_{n}\right)$ is updated.

5. Update all angle spectrum

Repeat steps (2)~(4) until the low-resolution image of all illumination angles is updated, which can be regarded as completing an iterative process.

6. Iteration to convergence

Repeat iteration steps (2)~(5) until the reconstruction algorithm converges, so as to obtain the high-resolution spectrum of the object, and then go back to the airspace by inverse Fourier transform to obtain the complex amplitude of the high-resolution object and complete the reconstruction process.

## 3. Proposed Method

**Figure 2:**
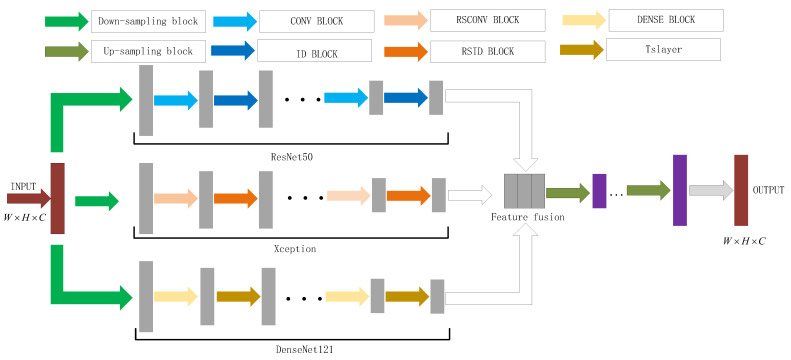
Deep multi-feature transfer network Fourier ptychographic microscopy imaging reconstruction model.

In this paper, we proposed a network model of deep multi-feature transfer network (DMFTN) Fourier ptychographic microscopy image reconstruction methods built using deep learning neural networks, which can simultaneously acquire the high-resolution intensity and phase of the sample, improve the quality of the FPM reconstruction problem, and shorten the reconstruction time. The DMFTN model is built, as shown in Figure 2, including: network model input, migration learning ResNet50 [32], Xception [33] and Densenet121 [34], down sampling feature extraction of the network framework, cascaded feature fusion strategy and upsampling reconstruction network structure.

### 3.1. Network Model Input

In the FPM system, hundreds of original low-resolution images are obtained simultaneously by programmable control of hundreds of LED array light sources, so the input data in the corresponding neural network is a three-dimensional image tensor with hundreds of channels. To extract features from the convolutional layer of a convolutional neural network, too many channels will cause the network parameters to increase exponentially. The convolutional layer will require hundreds or even thousands of channels, which is difficult for the network to implement. If the number of network input channels trained is fixed to the number of LEDs, the trained network will not work on other systems with different numbers of LEDs.

In order to avoid these problems, the low-resolution data of FPM are synthesized in the Fourier domain, and converted into a dual-channel complex amplitude image through the inverse Fourier transform as the input of the network [34]. This process can be expressed as:

(10) $$O_{n}(k)=F\left\{\sqrt{I_{nc}(r)}\right\}H(k+k_{n})+O_{n-1}(k)[1-H(k+k_{n})]$$

(11) $$o(r)=F^{-1}\left\{O_{LED}(k)\right\}$$

where n denotes the serial number of the image, the $\left(n=1,2,\ldots ,N_{LED}\right)$. o(r) is a complex form, and its intensity and phase are input to the neural network as a channel, respectively. Using Equations (10) and (11), the number of channels of input data is greatly reduced, while the spatial size is increased to contain more effective high-frequency information.

### 3.2. Reconstructed Network Structure of the Built Deep Multi-Feature Transfer Network

In this paper, DNN is used to reconstruct the network structure and train the model to learn the nonlinear mapping relationship between input and output. The original low-resolution intensity image is synthesized and input into the dual-channel complex amplitude through formulas (10) and (11) as the input of the network model data. After transfer learning ResNet50 [32], Xception [33] and Densenet121 [34] networks, the down sampling feature extraction is realized. The cascaded feature fusion strategy performs channel fusion on the image features extracted by the transfer learning network. Finally, the pre-upsampling reconstruction network module is used to reconstruct the high-resolution intensity and phase with the same size as the output size.

Through the training of data set, the network can achieve fast and high-quality FP reconstruction. From a functional point of view, it is mainly composed of four parts, constructing the residual network of the ResNet50 transfer learning network, the channel attention of the Xception transfer learning network, the dense connection of the DenseNet121 transfer learning network, and the pre-up sampling reconstruction network [35,36,37,38].

#### 3.2.1. Transfer ResNet50 Base-Layer

The neural network only needs to calculate the residual between input and output, and the output is obtained by adding the residual to the input. The residual network is very easy to implement, can greatly reduce the difficulty of network training and significantly improve the reconstruction effect. The ResNet50 transfer network is mainly composed of residual structures.

**Figure 3:**
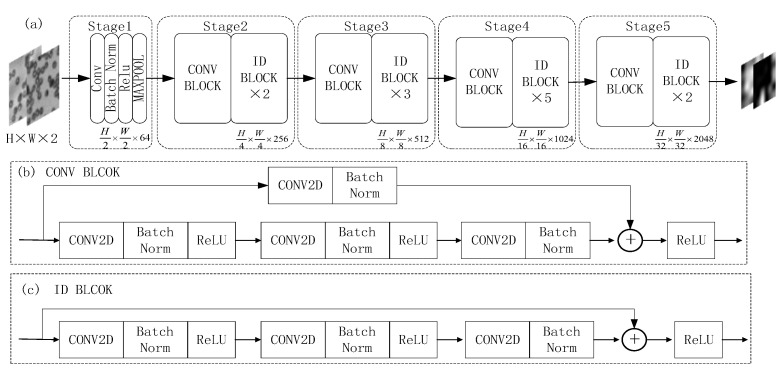
Migration ResNet50 base-layer network model framework: (**a**) schematic diagram of base-layer network model; (**b**) CONV BLOCK network structure module; (**c**) ID BLOCK network structure module.

As Figure 3 shows the transfer learning ResNet50 base-layer network model framework, where (a) represents the migration ResNet50 base-layer network model, (b) representation of the CONV BLOCK network structure module formed in the migration base layer and (c) represents the ID BLOCK network structure module formed in the migration-base layer.

#### 3.2.2. Transfer Xception Base-Layer

Different weights are applied to the feature information of different channels in the feature map to improve the utilization rate of effective information. Since different channels of the feature map correspond to different convolution kernel operations, by weighting specific channels, useful information can be more effectively combined, thereby improving the performance of the network. The Xception migration network is mainly composed of channel attention structure.

**Figure 4:**
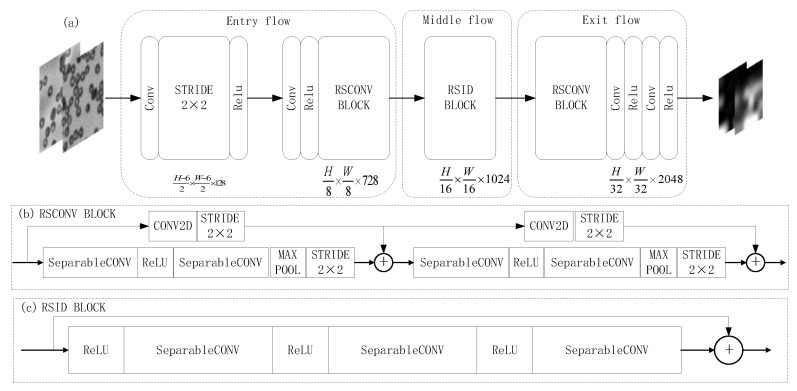
Migration Xception base network layer network model framework: (**a**) schematic diagram of the base network layer network model; (**b**) RSCONV BLOCK network structure module; (**c**) RSID BLOCK network structure module.

As Figure 4 shows the transfers learning Xception base-layer network model framework, where (a) represents the migration Xception base-layer network model, (b) represents the RSCONV BLOCK network structure module formed in the migration base-layer and (c) represents the RSID BLOCK network structure module formed in the migration base layer.

#### 3.2.3. Transfer DenseNet121 Base-Layer

Dense connection is established between multiple modules of the neural network, which greatly increases the number of paths of data flow. By introducing dense connections, each module is directly connected to the input and output, and each module is also connected to each other. The image features are reused to the greatest extent. The DenseNet121 migration network is mainly composed of dense connection block structure.

**Figure 5:**
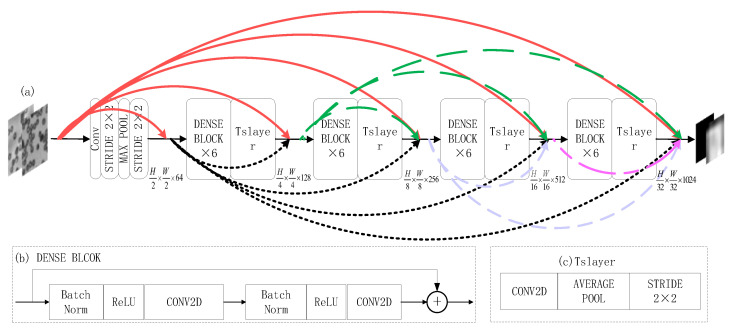
Migration of DenseNet121 base-layer network model framework: (**a**) schematic diagram of base-layer network model; (**b**) DENSE BLOCK network structure module; (**c**) Tslayer network structure module.

As Figure 5 shows the transfer learning DenseNet121 base-network model framework: where (a) represents the migrated DenseNet121 base-layer network model, (b) represents the DENSE BLOCK network structure module formed in the migration base layer and (c) represents the Tslayer network structure module formed in the migration base layer.

#### 3.2.4. Cascade Feature Fusion

In transfer learning, feature fusion can be used to connect multiple transfer network models together [34]. The purpose of feature fusion is to combine the features extracted from the image into a more discriminative feature than the input feature.

The feature fusion algorithm can fuse the output multiple feature maps to obtain the fused feature map, thus connecting multiple networks together, which is the fusion point. Multiple networks begin to learn independently before the fusion point, so when the feature fusion method is introduced, the network fuses the features of independent learning at the fusion point, and finally starts to learn together.

In this paper, the single feature extracted from three sub-network structures (ResNet50, Xception, DenseNet121) is trained for feature fusion. The feature fusion function retains the results of three feature maps, which makes the number of channels after fusion become the sum of the number of channels in the original feature map. The formula is:

(12) $$y=f\left(x^{a},x^{b},x^{c}\right)$$

(13) $$y_{i,j,2d}=x_{i,j,d}^{a},{\;y}_{i,j,2d-1}=x_{i,j,d}^{b},{\;y}_{i,j,2d+1}=x_{i,j,d}^{c}$$

where $x^{a},\;\;x^{b}{\;\mathrm{and}\;x}^{c}$ denote the characteristics of images obtained by different transfer neural networks, y represents the fused features. $y\in R^{H\times W\times D},H,\;\;W\;\mathrm{and}\;D$ represent the length, width and channel number of feature vector.

### 3.3. Pre-Upsampling

**Figure 6:**
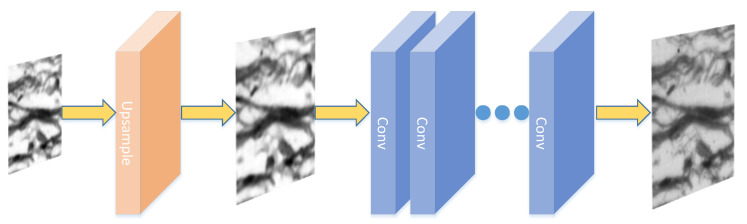
Pre-upsampling reconstruction module.

After feature fusion, the upsampling module is composed of convolution layer and pixel recombination layer to sample the feature map to the same spatial size as the network input. Through a convolution layer and a pixel recombination layer, the space size can be doubled, and the pixels are continuously convoluted upward until the pre-upsampling reconstruction module receives the dual-channel feature map output. The low-resolution image is up-sampled at the front end of the neural network. As shown in Figure 6, the blurred image can be made clear, and the operations of feature extraction, mapping and reconstruction are completed in the high-resolution space.

## 4. Experiment

The DMFTN reconstruction method proposed in this paper is verified on the simulation and experimental data sets. Using the simulation experimental data, the reconstruction results of the FPM reconstruction method under deep learning are compared and evaluated with the iterative phase recovery reconstruction alternating projection G-S [34] method, the latest Zhang [19] and the Zuo’s AS [39] method.

### 4.1. Experimental Environment

The test experimental platform is a computer with AMD Ryzen9 5900X CPU, 3.70 GHz, 64-bit operating system, 64G of memory and Window10 operating system. The programming language used to implement the method in this paper is python3.7, using the TensorFlow open-source framework; the G-S algorithm is run under the Matlab2018b environment. The Zhang et al. deep learning method uses the Keras open-source framework. The experimental reconstruction results were compared using the values of peak signal to noise rate (PSNR) and structural similarity (SSIM). Simulation and pre-processing of the dataset were implemented through MATLAB. For training the network, a learning rate of $4\times 10^{-5}$, Adaptive moment estimation (Adam) optimizer was used to implement gradient descent, the size of the input and truth data was 192 × 192 pixels, the loss function was Mean Square Error (MSE), and 200 epochs were trained on a graphics card with a training batch size is 4, the model is saved and the experiments are performed on the test experimental platform.

### 4.2. Construction of the Dataset

#### 4.2.1. Synthesis of Simulated Experimental Data

**Figure 7:**
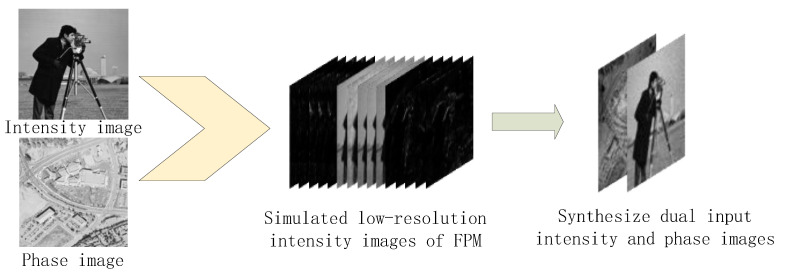
Process of simulating experimental data.

The photographer and street map are used as amplitude and phase images, respectively. The process of simulating experimental data is shown in Figure 7. In the simulation process, the NA of the objective lens is set to 0.13, the wavelength is set to 0.505 μm, and the simulated planar array light source is 13 × 13.169 low-resolution intensity images with 48 × 48 pixels were obtained by using the FPM imaging process. The dual-channel complex amplitude image was reconstructed as the model input through the traditional iterative phase reconstruction method. In order to simulate the influence of noise on the reconstruction results in the image acquisition process of the imaging system, the complex amplitude image input model with noise is reconstructed by adding Gaussian noise while obtaining low resolution in the FPM imaging process.

#### 4.2.2. Experimental Data Set Construction

**Figure 8:**
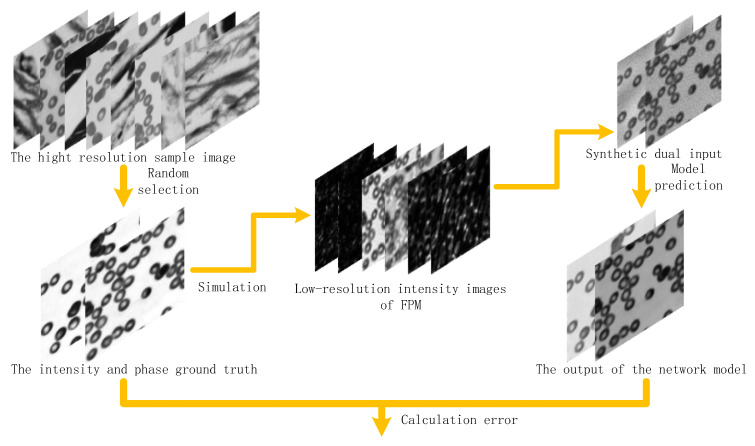
Construction of the dataset and training process of the network model.

In this paper, the simulation method is used to construct the data set [35], and the simulation image is directly used as the true value. The corresponding network model input data is generated by the simulation of the FPM imaging model, as shown in Figure 8. A total of 400 high-resolution images of various samples were collected, such as penile tissue slices, blood cell smears and alveolar tissue slices, etc. Through the random combination of phase and intensity, 1600 complex amplitudes were generated as the true value of the network output. These complex amplitudes are used to obtain low resolution intensity images through the FP imaging process, and the input method is synthesized to generate the input dual-channel intensity and phase of the network. In the process of image acquisition, the influence of natural light on the quality of image reconstruction provided by the experimental light source is considered. Therefore, Gaussian noise with mean of 0 and standard deviation is added to the input data to simulate the noise in the actual imaging process. The data generated by the simulation are randomly cut, and 25,600 groups of input and true value data are finally obtained. Among them, 90% are used as the network model training data set with the number of training sets being 23,040; 10% as the model test data set, and the number of test sets is 2560.

On the basis of the above simulation data set, in order to improve the processing effect of the network for real system acquisition data, a fine-tuning data set composed of real experimental equipment acquisition images is established. The original data of 50 groups of samples of Fourier laminated microscopy were collected, and the reconstructed image was used as the true value. Then, 450 groups of input low resolution images and corresponding true value images were obtained by random clipping, including 400 training sets and 50 test sets. In order to prevent the over-fitting of the network training process, the fine-tuning data set is randomly added to the simulation data set for training.

### 4.3. Evaluation Method of Reconstruction Results

The Mean Square Error (MSE) and accuracy index are used to measure the stability of the network model. Peak signal-to-noise ratio (PSNR) and structural similarity (SSIM) are used to evaluate the quality of image reconstruction.

#### 4.3.1. Evaluation Indicators of the Model

Loss function: The MSE loss function is used to evaluate the error between the predicted value and the real value of the sample. The stability of the model training is evaluated by analyzing the loss function value of the training sample value and the iteration number curve.

(14) $$MSE(y,y^{\prime })=\frac{\sum_{i=1}^{n}{\left(y_{i}-y_{i}^{\prime }\right)}^{2}}{n}$$

where y denotes the sample true value, $y^{\prime }$ denotes the sample predicted value and n denotes the number of iterations.

Accuracy: The number of correctly predicted samples divided by the number of all samples, which is generally used to evaluate the global accuracy of the model. The higher the accuracy, the closer the training accuracy curve is to the test accuracy curve and the better the model stability.

#### 4.3.2. Reconstructed Image Evaluation Index

Peak signal-to-noise ratio (PSNR): An objective criterion for evaluating the image, which indicates the quality of the output image compared with the original image after the image is processed. The larger the PSNR value, the better the image quality and the less the image distortion.

(15) $$PSNR=20\log_{10}(\frac{MAX_{I}}{\sqrt{MSE}})$$

where, $MAX_{I}$ denotes the maximum value of the image color, and the 8-bit sampling point is expressed as 255; MSE is the mean square error between the original image and the reconstructed image, and its definition can be expressed as:

(16) $$MSE=\frac{1}{mn}\sum_{i=0}^{m-1}{\sum_{j=0}^{n=1}\left\|I(i,j)-K(i,j)\right\|}^{2}$$

where, I denotes the original image, K denotes the reconstructed image and the size of the image is the same. m,n are the height and width of the image, respectively.

Structural similarity (SSIM) [40]: it is an evaluation index which is more consistent with human subjective evaluation and can be used to measure the similarity between digital images, which measures the image similarity in terms of brightness, contrast and structure, respectively, and is calculated as follows:

(17) SSIM(X,Y)=l(X,Y)c(X,Y)s(X,Y)

where, l(X,Y) comparing the brightness of two images, c(X,Y) comparing the contrast of two images, s(X,Y) comparing the structure of two images, are expressed as:

(18) $$l(X,Y)=\frac{2\mu_{{}_{X}}\mu_{Y}+C_{1}}{\mu_{X}^{2}+\mu_{Y}^{2}+C_{1}}$$

(19) $$c(X,Y)=\frac{2\sigma_{{}_{X}}\sigma_{Y}+C_{2}}{\sigma_{X}^{2}+\sigma_{Y}^{2}+C_{2}}$$

(20) $$s(X,Y)=\frac{\sigma_{{}_{XY}}+C_{3}}{\sigma_{X}\sigma_{Y}+C_{3}}$$

where, $\mu_{{}_{X}}$ and $\mu_{Y}$ denote the mean of the two images, $\sigma_{{}_{X}}$ and $\sigma_{Y}$ denote the standard deviation of the two images, $\sigma_{XY}$ are the covariance of the two images, $C_{1},C_{2},C_{3}$ are the constants of the calculation, in use, the parameters are generally set to $C_{3}=C_{2}/2$, to obtain the simplified form

(21) $$SSIM=\frac{\left(2\mu_{X}\mu_{Y}+C_{1})(2\sigma_{12}+C_{2}\right)}{\left(\mu_{1}^{2}+\mu_{2}^{2}+C_{1})(\sigma_{1}^{2}+\sigma_{2}^{2}+C_{2}\right)}$$

where $C_{1}={\left(0.01L\right)}^{2},C_{2}={\left(0,03L\right)}^{2}$, L is the dynamic range of the pixel value, SSIM takes the value range [0, 1], the larger the value, the smaller the image distortion.

### 4.4. Experimental Results and Analysis

#### 4.4.1. Network Model Evaluation

**Figure 9:**
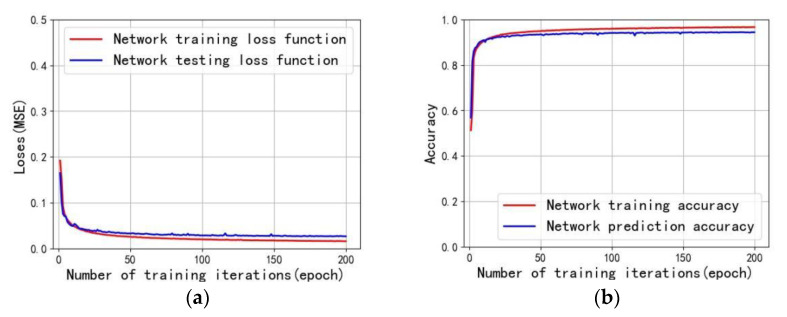
Evaluation of stability of model fitting curve. (**a**) Plot of training iterations vs. loss function; (**b**) plot of training iterations vs. accuracy.

In this paper, the stability of the network model is measured by the fitting curve of the loss function and accuracy index value of the training and test data output under different epochs, as shown in Figure 9. Figure 9a shows the curve of the number of training iterations and the loss function. As the number of iterations increases, the closer the fitting curve of the reconstruction network training loss function and the reconstruction network prediction loss function is to the model, the better the stability is. The prediction curve does not shake violently, and the model does not appear gradient explosion. Figure 9b is the curve of the number of training iterations and the accuracy rate. The closer the fitting curve of the reconstruction network training accuracy rate and the reconstruction network prediction accuracy rate is, the higher the predicted result value, and the better the reconstruction effect.

#### 4.4.2. Comparison of Reconstruction Performance under Noise Conditions

**Figure 10:**
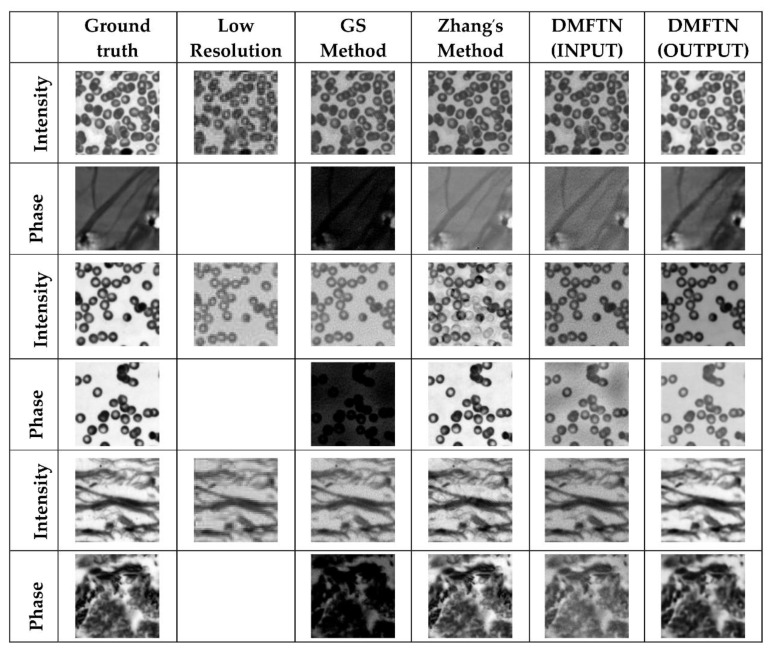
Reconstruction results under the same noise.

Considering the existence of Gaussian noise in the image acquisition process of Fourier ptychographic microscopy imaging system, the mean value is 0 and the standard deviation is Gaussian noise in the simulation test set. The peak signal-to-noise ratio (PSNR) and structural similarity (SSIM) are used to compare the reconstruction quality. The DMFTN method is compared with the traditional alternating projection method and the neural network method. As shown in Figure 10, Table 1 is the index value of different image reconstruction results under the same noise.

**Figure 11:**
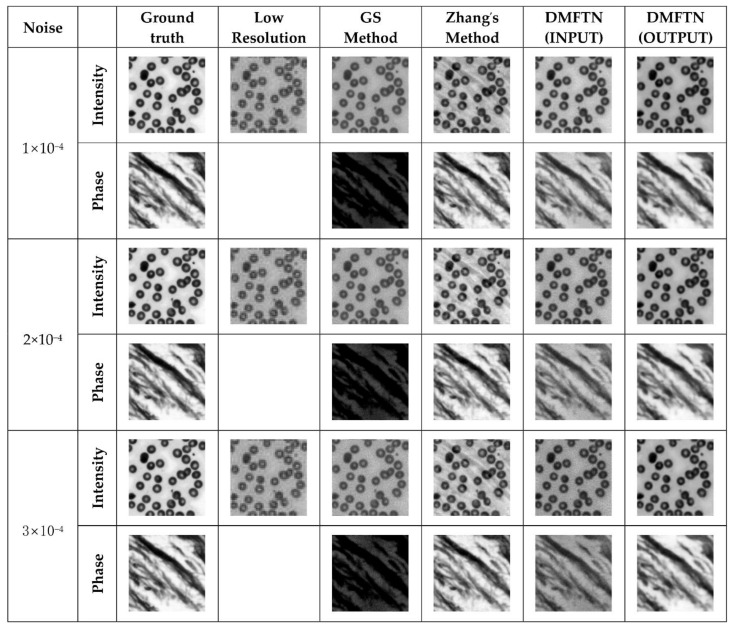
Reconstruction results under the different noise.

In order to verify the universality and robustness to noise of the reconstruction methods in this chapter, the reconstruction results of each method with different noise added under the same image are shown in Figure 11, and Table 2 shows the index values of the reconstruction results of the same image under different noise.

From the above experimental results, it can be seen that the reconstruction quality index values of DMFTN under different noise conditions are better than those of GS and Zhang et al. The DMFTN reconstruction results contain more details and less errors, while the GS method has poor phase reconstruction results, and the strength reconstruction results of Zhang et al. have artifacts.

#### 4.4.3. Reconstruction Performance with Reduced Amount of Acquired Image Data

In order to evaluate the performance of this method when the amount of image data is small, the reconstruction performance is tested when the amount of original collected image data is 9, 25, 49, 81, 121 and 169. The simulated light source of the image test data set is 9, 25, 49, 81, 121 and 169, and the Gaussian noise with the mean value of 0 and the standard deviation of $1\times 10^{-4}$ is added.

**Figure 12:**
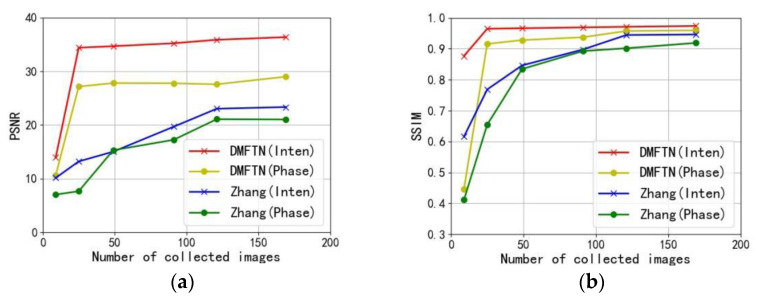
Reconstruction quality curve with reduced number of acquired images: (**a**) PSNR plot of the reconstruction results; (**b**) SSIM plot of the reconstruction results.

The test results are compared with the performance of Zhang et al.’s reconstruction methods, as shown in Figure 12, Figure 12a is the PSNR curve of the reconstruction results, and Figure 12b is the SSIM curve of the reconstruction results. The red and yellow represent the PSNR and SSIM curves of the reconstructed intensity and phase quality, and the blue and green represent the PSNR and SSIM curves of the reconstructed intensity and phase quality by Zhang et al.

It can be seen from the reconstructed quality curve that the intensity and phase PSNR curves of the reconstructed results of this method are higher than those of Zhang et al. The SSIM curve reconstructed by this method is higher and closer to the real value. Therefore, the use of deep learning method can not only improve the reconstruction quality, but also greatly improve the speed of image acquisition.

#### 4.4.4. Reconstruction Results on the Actual Dataset

**Figure 13:**
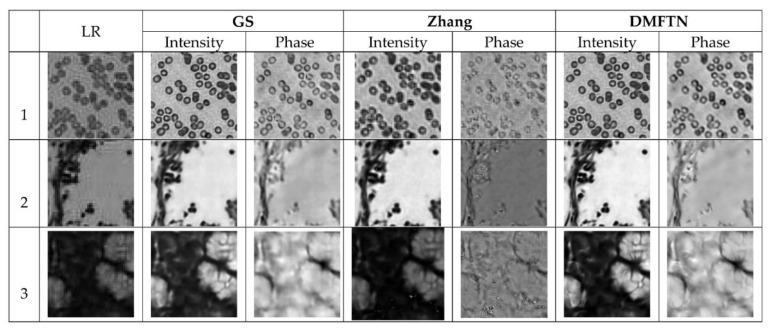
Reconstruction results on the actual dataset.

In order to verify the validity of Fourier ptychographic microscopy imaging reconstruction method based on deep multi-feature transfer network on real data, the test data set of fine-tuning data set constructed by equipment is used to compare the results of DMFTN with traditional phase recovery reconstruction and Zhang et al. Since the original low-resolution image can only be collected under experimental conditions, the true value of complex amplitude reconstruction results does not exist. Figure 13 shows the reconstruction results of DMFTN, GS and Zhang on the test set. It can be seen that the DMFTN performs well on real data, and obtains the optimal reconstruction results. The error of image background is less and the texture details are clearer.

#### 4.4.5. Reconstruction Time Comparison

The reconstruction process of FPM is the reconstruction hundreds of multi-angle illumination images to increase the number of acquired images to improve the redundancy of the reconstructed data and obtain high-resolution images, the FPM needs to consume a large amount of time resolution while improving the spatial resolution, resulting in the acquisition of the original image and the reconstruction process consuming a lot of time. Therefore, in the reconstruction process, not only the reconstruction quality but also the reconstruction speed should be considered.

When the image pixel size is 192 × 192, the DMFTN reconstruction method proposed in this paper is compared with the traditional phase recovery method (GS), adaptive step reconstruction method (AS) and the convolution neural network reconstruction method proposed by Zhang et al. Since DMFTN is based on deep learning without iterations, it has the least reconstruction time, as shown in Table 3.

## 5. Summary

In this paper, a multi-convolutional feature fusion network Fourier ptychographic microscopy imaging reconstruction model (DMFTN) is proposed for FPM reconstruction, which realizes FPM reconstruction under deep learning. The method uses ResNet50, Xception and DenseNet121 migratory learning network frameworks, a cascaded feature fusion strategy and a pre-upsampling reconstruction network to construct a DMFTN model; the multi-convolution migratory learning network is used to extract the complementarity of feature information and improve the utilization rate of feature fusion. The pre-upsampling reconstruction network improves the details of high-resolution image reconstruction. The experiments verify that the trained FPM neural network can reconstruct higher quality intensity and phase images with faster speed; the method has higher robustness to noise and blurring and other factors, which can greatly improve the temporal resolution of FPM and promote its practical application. However, the use of deep learning to achieve FPM reconstruction will also have limitations. The limitations come from the construction of data sets. If the experimental equipment is used to obtain the data set, there is no true value of the data set. If the simulation data sets are obtained under ideal conditions, the distortion from the system is not considered.

Moreover, due to the rapid development of powerful tools such as TensorFlow and other deep learning tools optimizers and loss functions, it is possible to combine new these techniques to obtain further improvements, and to replace some layers of DMFTN with super-resolution medium and high-level architectures for better model reconstruction.

## Figures and Tables

**Table 1:** Index values of different image reconstruction results under the same noise.

$\mathrm{Add}\;\mathrm{the}\;\mathrm{Noise}\;\mathrm{Level}.\;3\times 10^{-4}$		Low Resolution (PSNR/SSIM)	G-S H-Method (PSNR/SSIM)	Zhang’s Method (PSNR/SSIM)	DMFTN (INPUT) (PSNR/SSIM)	DMFTN (OUTPUT) (PSNR/SSIM)
Group 1 Images	Intensity	21.25/0.723	24.02/0.761	26.27/0.922	23.91/0.771	**34.79/0.979**
	Phase		13.93/0.278	22.52/0.786	12.12/0.083	**25.99/0.965**
Group 2 Images	Intensity	24.76/0.844	22.62/0.592	20.03/0.787	23.13/0.613	**35.73/0.981**
	Phase		4.29/0.229	16.01/0.877	3.51/0.016	**33.43/0.976**
Group 3 Images	Intensity	24.77/0.840	28.94/0.554	22.77/0.920	23.66/0.678	**36.54/0.979**
	Phase		10.93/0.264	21.52/0.932	9.19/0.015	**24.84/0.932**

**Table 2:** Index values of the same image reconstruction results under different noise.

Same IMAGE		Noise Level	Low Resolution (PSNR/SSIM)	I-S Method (PSNR/SSIM)	Zhang’s Method (PSNR/SSIM)	DMFTN (INPUT) (PSNR/SSIM)	DMFTN (OUTPUT) (PSNR/SSIM)
Group 1 Images	Intensity	$1\times 10^{-4}$	24.42/0.827	28.85/0.848	30.38/0.861	28.76/0.937	**35.12/0.981**
	Phase			7.97/0.223	26.11/0.928	5.99/0.021	**26.63/0.45**
Group 2 Images	Intensity	$2\times 10^{-4}$	22.24/0.718	25.12/0.722	27.62/0.836	25.77/0.737	**34.99/0.979**
	Phase			7.43/0.205	25.72/0.923	6.17/0.118	**26.35/0.944**
Group 3 Images	Intensity	$3\times 10^{-4}$	20.11/0.622	23.26/0.643	21.47/0.861	23.91/0.662	**34.79/0.979**
	Phase			7.27/0.197	18.78/0.92	5.96/0.013	**26.08/0.952**

**Table 3:** Comparison of reconstruction time of four methods.

Reconstruction Method	Number of Iterations	Reconstruction Time	Multiple
**G-S Method**	50	1.684 s	17
**AS Method**	50	1.977 s	22
**Zhang’s Method**	20	204.5 s	48,822
**Method of this article (** **DMFTN** **)**	**0**	**0.091 s**	**1**

## Data Availability

Not applicable.
